# Advanced Robotic Therapy Integrated Centers (ARTIC): an international collaboration facilitating the application of rehabilitation technologies

**DOI:** 10.1186/s12984-018-0366-y

**Published:** 2018-04-06

**Authors:** Hubertus J. A. van Hedel, Giacomo Severini, Alessandra Scarton, Anne O’Brien, Tamsin Reed, Deborah Gaebler-Spira, Tara Egan, Andreas Meyer-Heim, Judith Graser, Karen Chua, Daniel Zutter, Raoul Schweinfurther, J. Carsten Möller, Liliana P. Paredes, Alberto Esquenazi, Steffen Berweck, Sebastian Schroeder, Birgit Warken, Anne Chan, Amber Devers, Jakub Petioky, Nam-Jong Paik, Won-Seok Kim, Paolo Bonato, Michael Boninger, for the ARTIC network, Eric Fabara, Catherine Adans-Dester, Jean O’Brien Murby, Lori Laliberte, Gadi Revivo, Stella Lee, Theresa Toczylowski, Kay Fei Chan, Seng Kwee Wee, Pang Hung Lim, Wei Sheong Lim, Juliana Yun Ying Wang, Wing Kuen Lee, Chui Ni Ong, Cheng Hong Ong, Charlene Cheryl Pereira, Siew Yee Lee, Alexander Dewor, Michael Urban, Tabea Aurich, Anja Lucic, Thomas Nastulla, Katharina Badura, Josephine Steinbichler, Myungki Ji, Yunsung Oh, Salvatore Calabro, Leslie van Hiel, Martina Spiess, Lars Lünenburger, Gery Colombo, Irin Maier

**Affiliations:** 1 Rehabilitation Center for Children and Adolescents, University Children’s Hospital Zurich, Mühlebergstrasse 104, CH-8910 Affoltern am Albis, Switzerland; 2 Department of Physical Medicine and Rehabilitation, Harvard Medical School, at Spaulding Rehabilitation Hospital, Charlestown MA, USA; 3 University College Dublin, Dublin, Ireland; 4 Acute Neurological Rehabilitation Unit, Wellington Hospital, London, UK; 5 Shirley Ryan AbilityLab, Chicago, USA; 6 Tan Tock Seng Hospital Rehabilitation Centre, Singapore, Republic of Singapore; 7 Rehaklinik Zihlschlacht, Center for Neurological Rehabilitation, Zihlschlacht, Switzerland; 8 Department of Physical Medicine and Rehabilitation, MossRehab, Philadelphia, USA; 9 Clinic for Neuropediatrics and Neurological Rehabilitation, Epilepsy center for children and adolescents, Schön Klinik Vogtareuth, Vogtareuth, Germany; 10 Paediatric Neurology, Developmental Medicine and Social Paediatrics, Ludwig Maximilian University, Hauner Children’s Hospital, Munich, Germany; 11 Sheltering Arms Physical Rehabilitation Center, Richmond, USA; 12 Rehabilitation Centre Kladruby, Kladruby, Czech Republic; 13 Department of Rehabilitation Medicine, Seoul National University Bundang Hospital, Seongnam, Republic of Korea; 14 Department of Physical Medicine and Rehabilitation, University of Pittsburgh and VA Pittsburgh Health Care System, Pittsburgh, USA

## Abstract

**Background:**

The application of rehabilitation robots has grown during the last decade. While meta-analyses have shown beneficial effects of robotic interventions for some patient groups, the evidence is less in others. We established the Advanced Robotic Therapy Integrated Centers (ARTIC) network with the goal of advancing the science and clinical practice of rehabilitation robotics. The investigators hope to exploit variations in practice to learn about current clinical application and outcomes. The aim of this paper is to introduce the ARTIC network to the clinical and research community, present the initial data set and its characteristics and compare the outcome data collected so far with data from prior studies.

**Methods:**

ARTIC is a pragmatic observational study of clinical care. The database includes patients with various neurological and gait deficits who used the driven gait orthosis Lokomat® as part of their treatment. Patient characteristics, diagnosis-specific information, and indicators of impairment severity are collected. Core clinical assessments include the 10-Meter Walk Test and the Goal Attainment Scaling. Data from each Lokomat® training session are automatically collected.

**Results:**

At time of analysis, the database contained data collected from 595 patients (cerebral palsy: *n* = 208; stroke: *n* = 129; spinal cord injury: *n* = 93; traumatic brain injury: *n* = 39; and various other diagnoses: *n* = 126). At onset, average walking speeds were slow. The training intensity increased from the first to the final therapy session and most patients achieved their goals.

**Conclusions:**

The characteristics of the patients matched epidemiological data for the target populations. When patient characteristics differed from epidemiological data, this was mainly due to the selection criteria used to assess eligibility for Lokomat® training. While patients included in randomized controlled interventional trials have to fulfill many inclusion and exclusion criteria, the only selection criteria applying to patients in the ARTIC database are those required for use of the Lokomat®. We suggest that the ARTIC network offers an opportunity to investigate the clinical application and effectiveness of rehabilitation technologies for various diagnoses. Due to the standardization of assessments and the use of a common technology, this network could serve as a basis for researchers interested in specific interventional studies expanding beyond the Lokomat®.

## Background

The number of technological devices that therapists can utilize to treat people with neurological impairments has grown substantially during the last decade. Alongside this growth in clinical use, research involving robotic therapy has grown rapidly. A search in Pubmed with the terms “robot” OR “robotic*” AND “rehabilitation” revealed 2225 hits (March 2017) with research markedly increasing after 2010. Despite this increase in research activity and clinical use, the effectiveness of robot-assisted interventions in neurorehabilitation is still in debate. While in some patient populations, for example adults with stroke, meta-analyses have shown that robotic interventions for the lower and upper extremity can be beneficial [1, 2], current evidence is much less convincing in other patient groups, such as spinal cord injury (SCI), traumatic brain injury (TBI), multiple sclerosis (MS) and cerebral palsy (CP).

When comparing the effectiveness of robot-assisted gait training (RAGT) to conventional interventions of similar dosage in adult patients after SCI, it appears that neither intervention is superior [3, 4]. In other populations, such as MS, a small number of pilot studies have been conducted, and a review [5] concluded that the evidence for the effectiveness remained inconclusive. In adult patients with TBI, to our knowledge, there is only one randomized controlled trial that investigated the effectiveness of RAGT [6]. While RAGT improved gait symmetry compared to manually assisted body-weight supported treadmill training, improvements in other gait parameters were not different between the interventions. In children with CP, the body of evidence is similarly small, as only two randomized trials were found [7, 8]. To the authors’ knowledge, there are no randomized controlled trials in children with other diagnoses. Studies comparing effectiveness between different patient groups are lacking.

One important factor leading to the lack of conclusive research is the relatively small number of available centers and participating patients and consequently the small statistical power of attempted studies. Multicenter collaborations are needed to achieve adequate number of participants. Several of the limitations in the evidence of the application of RAGT arise from patient selection criteria and use of different, poorly described and/or low-dosed training protocols. For example, when systematically reviewing the literature in children, we found no paper describing a training protocol on how to apply a robot for rehabilitation of gait [9]. Most of the systematic reviews mentioned that it is extremely difficult to pool results from studies due to the large variability in treatment duration and frequency, contents of the training and inclusion criteria of the patients. For children with CP, an expert team was created to formulate goals, inclusion criteria, training parameters and recommendations on including RAGT in the clinical setting, to assist therapists who train children with CP with the Lokomat® (Hocoma AG, Volketswil, Switzerland) [9]. Such information could be used as a first step in defining training protocols, but this information is missing for most other patient groups.

While randomized controlled trials are usually considered the “gold standard” in building solid evidence in the field of medicine, it is often difficult for rehabilitation specialists working in the clinical environment to interpret the findings with respect to the population of patients they treat on a daily basis. Randomized controlled trials require a specialized team, a controlled setting and a strict selection of patients according to well defined inclusion and exclusion criteria. These criteria often select individuals most likely to benefit based on specific parameters and lack of co-morbidities. These narrow criteria may impact the ecological validity, as results only apply to a minority of patients. This was recently investigated by Dörenkamp et al. [10] who reported that the majority of patients in primary care (40% at the age of 50 years and at least two-thirds of the octogenarian population [11]) simultaneously suffered from multiple medical problems. Further, improvements in function might be less comparable to results described in randomized controlled trials and the treatment regimens used may not be applicable to patients with multiple comorbidities.

To overcome these issues, we established the Advanced Robotic Therapy Integrated Centers (ARTIC) network to collect data from patients using RAGT in a wide variety of clinical settings. ARTIC hopes to develop guidelines for usage as well as to answer scientific questions concerning the use of RAGT. While the ARTIC network includes a general patient population, other research networks focus on a specific disorder or diagnostic group (see, for example [12, 13]). ARTIC focuses on a common technological intervention – currently the driven gait orthosis Lokomat® – and aims to gather evidence for the efficient and effective use of robotic therapy. Variation in practice among ARTIC members together with collection of common data and outcome measurements will enable the group to draw strong, generalizable conclusions. Further goals include establishing standardized treatment protocols and increasing medical and governmental acceptance of robotic therapy. The aims of this paper are to introduce the ARTIC network to the clinical and research community, present initial data on the characteristics of included patients and compare these to those known from existing epidemiological data and interventional studies.

## Methods

### The ARTIC network

ARTIC is an international group of diverse, clinically renowned centers (Table 1) whose goal is to advance the science of rehabilitation robotics. ARTIC includes clinicians who use the robotics and researchers who can evaluate that usage and learn from the diversity of practice as well as patient populations. ARTIC receives technical and administrative support from a manufacturer. ARTIC uses a prospective observational research design referred to as practice-based evidence. Network members treat different patient groups (Table 1) of different ages in various in- or out-patient settings.

**Table 1:** Members of the ARTIC network

Institutions	Patients / year^a^	Patient diagnoses	Lokomat® since	Other technologies
Spaulding, Boston, USA	50	SCI, CP, stroke, TBI	2006	ArmeoSpring, ArmeoBoom, Erigo, FES bikes, ReWalk, Ekso, Bioness H200, Bioness L300 plus
Shirley Ryan AbilityLab^b^, Chicago, USA	30	SCI, CP	2007	ArmeoSpring, Intelligent Stretcher
MossRehab, Philadelphia, USA	20	SCI, TBI, stroke	2008	ArmeoSpring, ArmeoPower, Amadeo, Andago, Diego, G-eo, Reo, ReWalks, Safe Gait, Pablo, Tibion, Tymo,
Sheltering Arms Physical Rehabilitation Center, Richmond, USA	200	SCI, TBI, stroke, MS	2011	Zero G, FES bikes, Bioness H200 and L300+, Balance Master, Alter G Bionic Leg, ArmeoPower, SAEBO, SAEBO MAS, Reo Go
Wellington Hospital, London, UK	30	SCI, TBI, stroke	2011	ArmeoSpring, Erigo, Erigo Pro, FES bike, Bioness H200, Indego
Tan Tock Seng Hospital Rehabilitation Centre, Singapore, Republic of Singapore	20	SCI, TBI, stroke, CP	2008	ArmeoSpring, ArmeoBoom, NeuroMove, Handtutor, Jintronix Gaming platform, Dynavision, MIT Manus (wrist robot), ReJoyce, SMART Balance Master
Schön Klinik, Vogtareuth, Germany	25	SCI, TBI, stroke, CP	2006	ArmeoSpring, Biometrix, RehaMove
Von Haunersches Kinderspital München, München, Germany	30	CP	2006	–
Rehaklinik Zihlschlacht, Center for Neurological Rehabilitation, Zihlschlacht, Switzerland	40	SCI, stroke, TBI, Parkinson, MS	2006	Erigo FES, Andago, ArmeoSpring, ArmeoPower, ARMin, Bioness L300 Plus, Valedo, VICTOR and EMMA, Tipstim
Rehabilitation Center for Children and Adolescents, University Children’s Hospital Zurich, Affoltern am Albis, Switzerland	35	SCI, CP, stroke, TBI	2005	Andago, Amadeo, ArmeoSpring, ChARMin, Diego, Erigo, Myro, YouGrabber
Rehabilitation Centre Kladruby, Kladruby, Czech Republic	80	SCI, TBI, Stroke	2009	ArmeoPower, ReoGo, Gloreha glove Professional, Ekso, ErigoPro, Hand Tutor, FES bike, Balance Manager, Balance master, Thera Trainer Balance, Postural treadmill with dynamic weightless system
Seoul National University Bundang Hospital, Seongnam, Republic of Korea	40	Stroke, TBI, SCI	2015	ArmeoPower
University of Pittsburgh Medical Center, Pittsburgh, USA	Currently consultant			
Shepherd, Atlanta, USA	Currently consultant			

*Abbreviations*: *SCI* Spinal Cord Injury, *CP* Cerebral Palsy, *TBI* Traumatic Brain Injury, *MS* Multiple Sclerosis. ^a^Number of patients expected to be contributed to the database annually; ^b^previously known as Rehabilitation Institute of Chicago

As an approach to begin this ambitious international multicenter undertaking, it was essential to start with a single common device, with the intention of learning and expanding to other devices. At initial meetings, ARTIC members discussed multiple rehabilitation robotic devices and decided to go with the Lokomat® because it was the most widely used device with, at that time, over 550 worldwide devices in use since its market introduction in 2000 [14] and all but one of the founding centers had active clinical programs using the Lokomat®.

Careful mechanisms were put in place to assure scientific independence from the industrial partner. For example, ARTIC members elect the leadership independently, and the ARTIC database is located at an independent medical center (Boston) and governed by that center’s human subject regulations.

The centers communicate with each other on a regular basis. We have monthly phone and internet conferences for the therapists, who update each other on new developments, discuss issues with the database or propose solutions for improving the clinical application of the Lokomat®. The principal investigators of each center have at least quarterly calls and meet in person once a year. Criteria to become part of the network are: at least one person who speaks English, site-specific IRB approval, able to join the web/phone conferences, able to contribute at least 20 complete datasets per year (definition see paragraph “Database”), therapists with advanced training in technology application, and sufficient expertise in applying the robotic system (i.e. more than 700 h of Lokomat® training).

### Common technological intervention: The Lokomat®

**Fig. 1:**
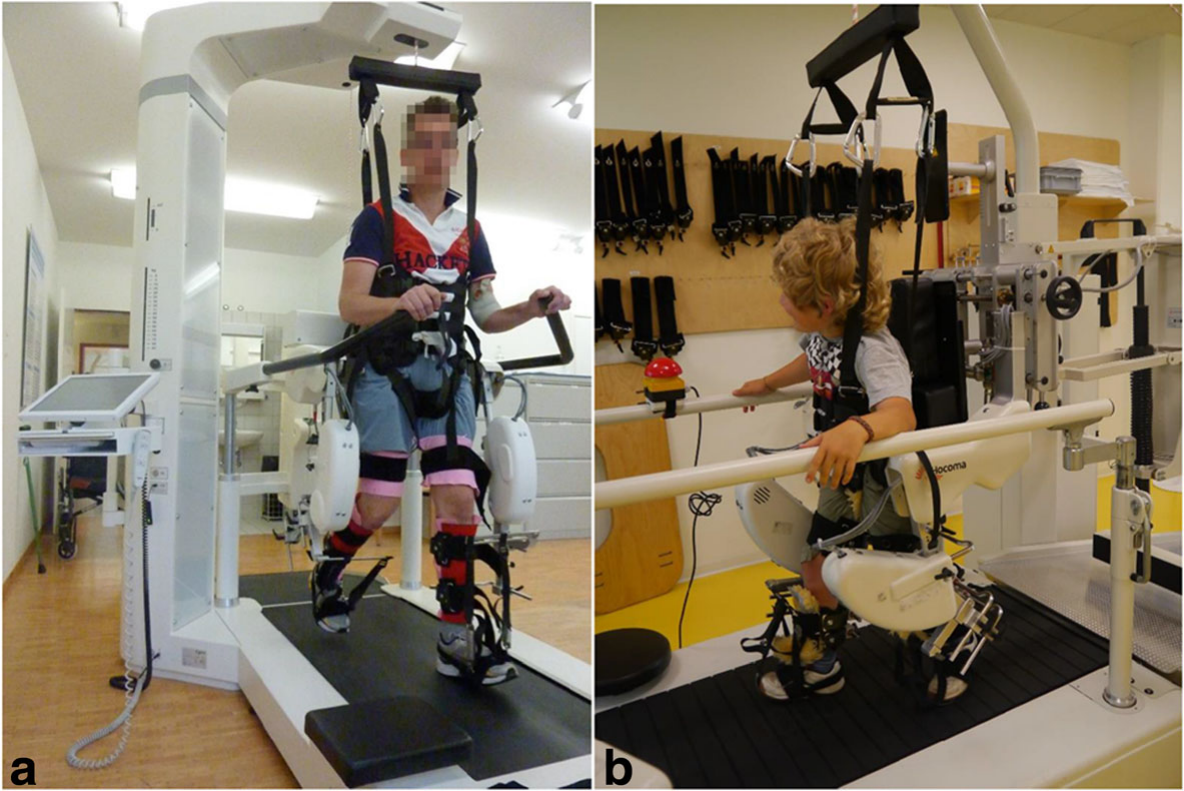
Lokomat® system (of different generations) with (**a**) adult leg orthoses and (**b**) pediatric leg orthoses. Patients walk on a treadmill belt, are weight supported, and the exoskeleton device guides the legs through a physiological walking pattern

The Lokomat® exoskeleton system comprises a treadmill belt, a weight support system and a driven gait orthosis for both legs (Fig. 1). Depending on the size of the patient, there are pediatric leg orthoses for children with a femur length between 21 and 35 cm, or adult leg orthoses intended for patients with a femur length between 35 and 47 cm. The patient is secured with three cuffs per leg to the orthosis. The hip and knee joint of the device are actuated. The robotic control uses an adjustable (impedance) controller with adjustable pre-defined trajectories for hip and knee joints. Elastic straps provide dorsiflexion assistance to the feet. The Lokomat® system can be adjusted to get the best possible fit for each patient. Therapists can use games providing biofeedback for increasing patients’ motivation (see e.g. [15, 16]). Recent developments include a FreeD mode (moveable pelvis and leg cuffs), new control mechanisms (i.e. path control rather than position control) and innovative virtual scenarios.

Inclusion criteria for Lokomat® training are a femur length of at least 21 cm (pediatric Lokomat®) or 35 cm (adult Lokomat®), a bodyweight ≤135 kg, and being able to signal pain, fear or discomfort reliably. Patients unable to use the Lokomat® are those who have severe lower extremity contractures, fractures, osseous instabilities, osteoporosis, severe leg length discrepancy, unhealed skin lesions, acute thrombo-embolic disease, cardiovascular instability, mechanical ventilation, severe cognitive deficits, or aggressive and self-harming behavior.

### Database

**Fig. 2:**
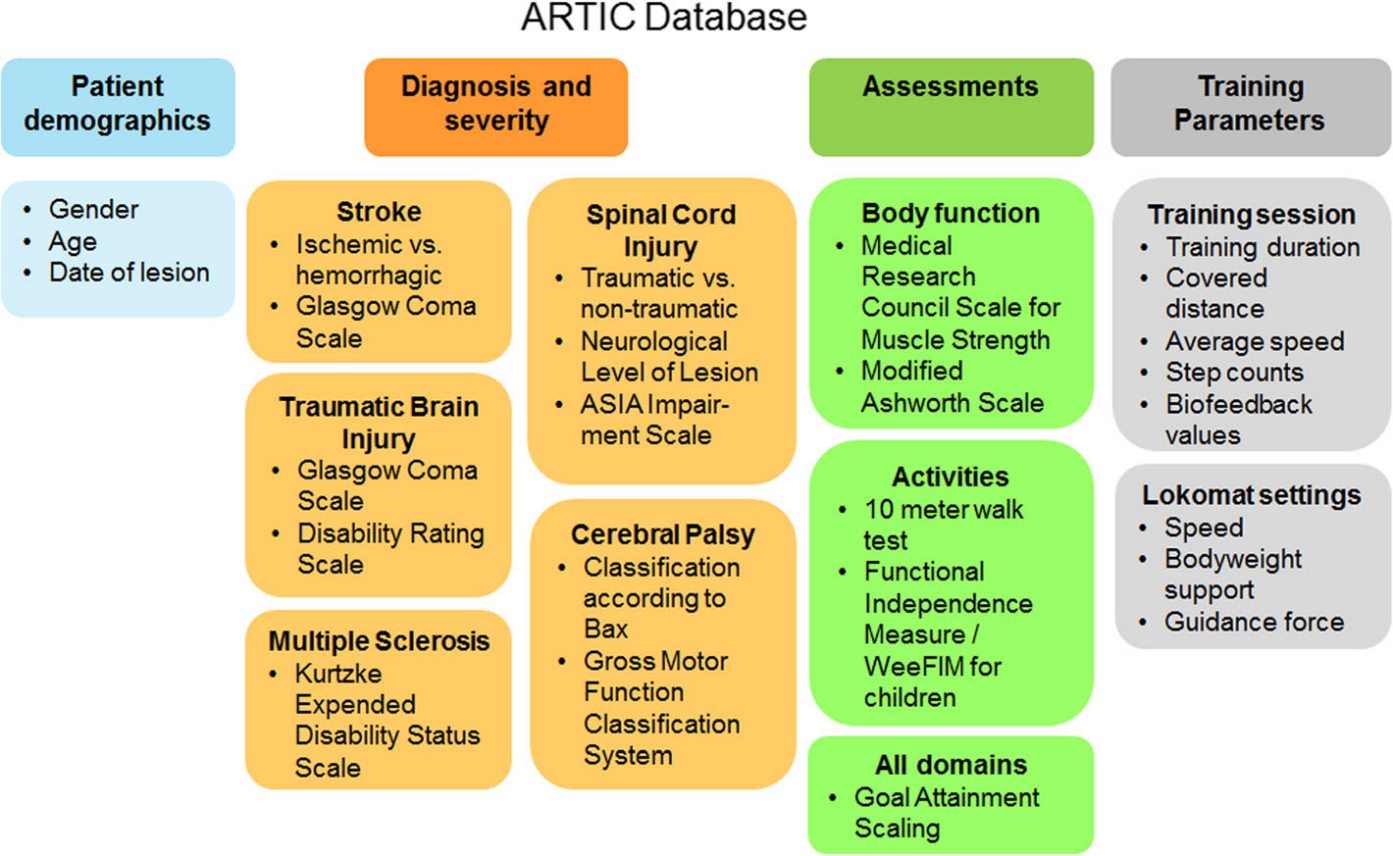
ARTIC Database containing information on patient demographics, diagnosis and severity, assessments and training parameters. Training parameters are stored for each separate training session to enable an evaluation over time. The 10-Meter Walk Test and the Goal Attainment Scaling are two core clinical assessments

One core component of the network was the development of the online database. The international multicenter approach of this project ensures the ascertainment of a sufficient number of cases with a broad spectrum of neurological and functional deficits. For each patient, gender, age, and date of the lesion (for acquired lesions) are recorded along with diagnosis-specific indicators of the severity and basic characteristics of the disorder (Fig. 2). For example, while for patients with SCI the ASIA Impairment Scale (AIS) and neurological level of lesion are assessed [17], the classification according to Bax [18] and the Gross Motor Function Classification System (GMFCS) are collected for children with CP [19].

The selected assessments cover several domains according to the International Classification of Functioning, Disability, and Health (ICF; e.g. body functions, activities, participation). We strived for a good balance between the time and effort required and the amount of collected information, as the goal was to have centers implement ARTIC activities into clinical routines with existing clinical personnel and resources.

The core clinical assessment set consists of the 10-Meter Walk Test (10MWT) and the Goal Attainment Scaling (GAS). The 10MWT was originally developed to measure walking capacity in patients with stroke [20] and was shown to be valid and reliable in a mixed group of neurological patients [21]. Its psychometric properties have been well investigated in adult patients with incomplete spinal cord injury showing good reliability and excellent responsiveness [22, 23]. In these patients, walking speed could also predict functional walking performance well [24]. In adult patients with stroke, the 10MWT proved sensitive to change (although the 5-m walk test was slightly better) [25]. In children with neurological gait disorders, the 10MWT showed good reliability [26].

The GAS is an example of an individualized evaluative instrument, which is used for measurement of changes in individual patients and groups of patients based on self-selected goals. Originally, a 5-point scale was developed by Kiresuk and Sherman [27], but we are using the more recent 6-point scale according to Steenbeek et al. [28]. The following scores apply: − 3, worse than start; − 2, equal to start; − 1, less than expected; 0, expected goal; + 1, somewhat more than expected, and + 2, much more than expected. The GAS proved applicable to patients with acquired brain lesions [29]. In pediatric rehabilitation, the GAS has been shown to be reliable, as the scales constructed by the children’s therapists had an inter-rater reliability of .82 (95% confidence interval .73–.91) [30], and the sensitivity to change proved to be good (for a review article see [31]). Also for adult patients, a review supported the validity, reliability and sensitivity of goal setting [32]. In the ARTIC database, there is an option to indicate whether the defined goals reflect an aspect of gait speed, endurance, or quality or other gait-related aspects.

Additional assessments that can be recorded in the database include the Modified Ashworth Scale (MAS), which is commonly used as an ordinal measure of spasticity [33] and the Medical Research Council Scale muscle strength score, which is commonly used and reliable [34]. Muscle strength is scored between 0 (total paralysis) to 5 (normal; active movement, full range of motion against gravity, and full resistance in a functional muscle position expected from an otherwise unimpaired person). Centers that use the Functional Independence Measure (FIM) [35] or the Functional Independence Measure for children (WeeFIM) [36] also enter these in the database.

Besides clinical and functional information, data from each Lokomat® session are automatically collected and can subsequently be integrated into the database (Fig. 2). This study concept allows for a detailed evaluation of training intensity, frequency, and parameters in comparison to therapy progress and outcome taking into account a basic characterization of the neurological condition of each patient.

In this paper, we used only descriptive analyses.

## Results

**Fig. 3:**
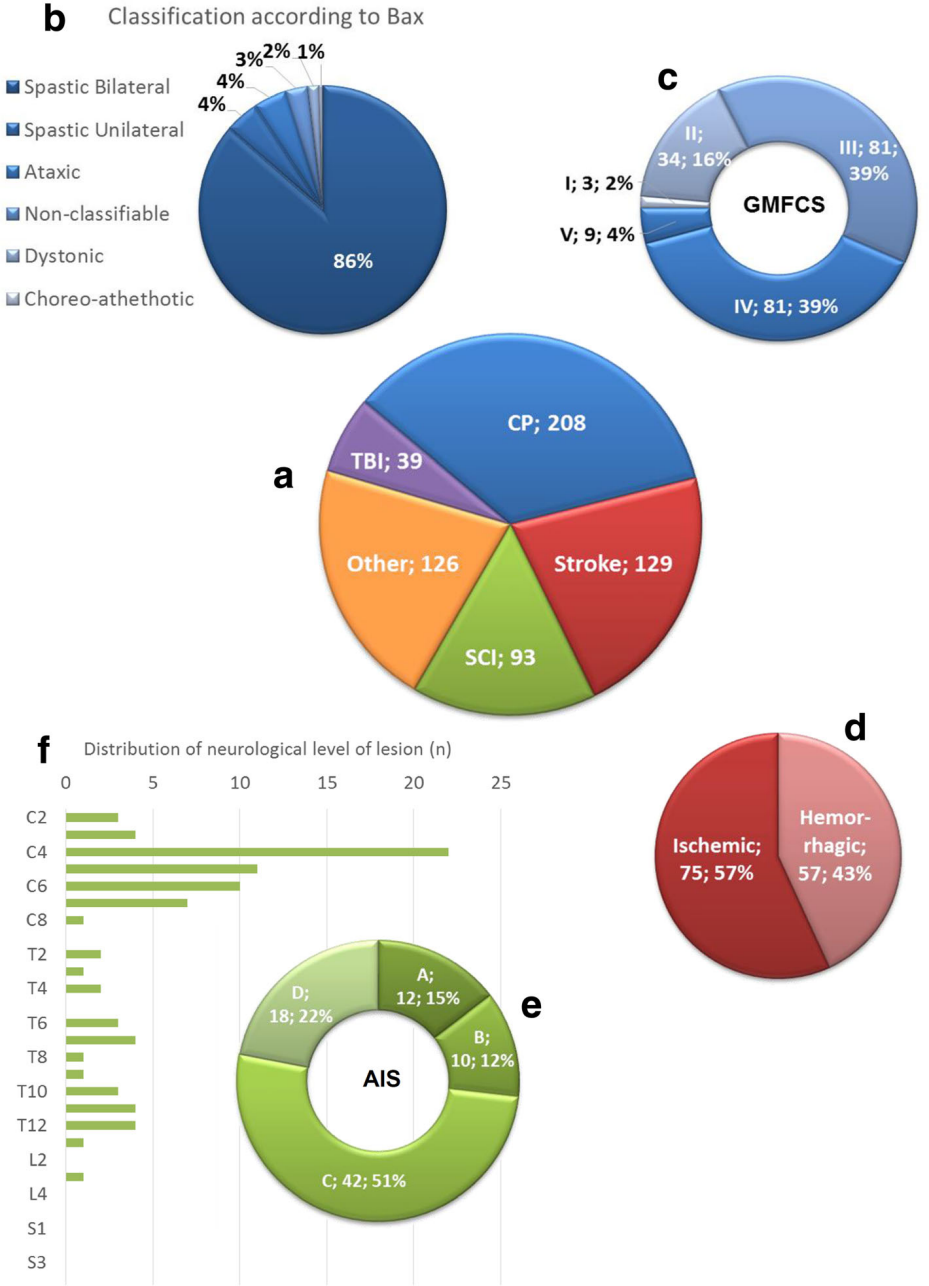
Clinical characteristics of the patients. (**a**) The largest group of patients are those with a Cerebral Palsy (CP), followed by stroke, spinal cord injury (SCI) and traumatic brain injury (TBI). (**b**) The clinical diagnosis according to Bax and (**c**) the severity grade according to the Gross Motor Function Classification System (GMFCS) are shown for patients with CP. (**d**) For patients with stroke, the distribution between patients with ischemic versus hemorrhagic stroke is shown. (**e**) For patients with SCI, the ASIA Impairment Scale (AIS) is shown and reflects the severity (AIS A, sensorimotor complete; AIS B motor complete, sensory incomplete; AIS C and D sensorimotor incomplete with AIS C indicating that less than half of the muscles below the neurological level of lesion have a muscle grade of 3 or more and AIS D indicating that half or more of the muscles below the neurological level of lesion have a grade 3 or higher). (**f**) Finally, the distribution of the neurological level of lesion is presented. Abbreviations: C, cervical; T, thoracic; L, lumbar and S, Sacral

Data collection started in January 2014. Several centers joined later (e.g. Vogtareuth, Munich, Wellington, Prague, Bundang). The number of patients entered into the ARTIC database by May 2016 was 595. The four largest groups based on diagnoses were CP, stroke, SCI, and TBI. Another large group, “others”, included patients with various other neurological diagnoses, such as Guillain-Barré syndrome, brain tumors, encephalitis, transverse myelitis, myelomeningocele, and others (Fig. 3).

**Fig. 4:**
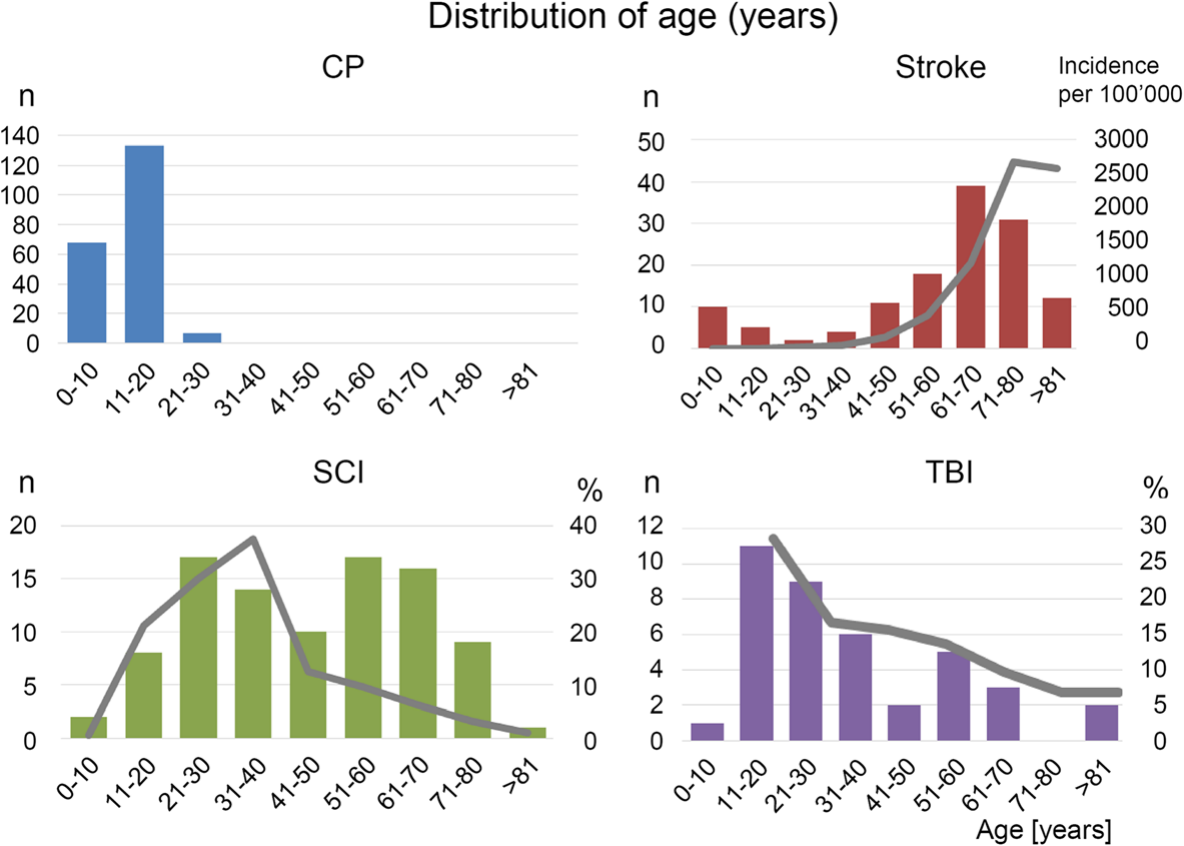
Histograms showing the distribution of age for patients with Cerebral Palsy (CP), stroke, spinal cord injury (SCI), and traumatic brain injury (TBI). Please note the different scales on the y-axes. Grey lines indicate distributions of age, which are based on numbers from the following epidemiological studies: stroke [40], SCI [53], and TBI [61]

To provide insight into the type of patients receiving Lokomat® therapy, we have presented more details of the largest patient groups. While the distribution of gender was relatively similar for patients with CP (females 45%) and stroke (females 46%), we found more males in patients with SCI (72%) and TBI (77%). Furthermore, the distribution of age was different between the groups (Fig. 4). Patients with CP were mainly under the age of 20. For patients with SCI the age showed a close-to-normal distribution, although we observed some peaks between 21 and 30 and 51–60 years. While patients with stroke showed a skewed distribution to the right, with a peak between 60 and 70 years, patients with TBI showed a skewed distribution to the left with a peak between 11 and 20 years.

We also evaluated the time from injury to starting their first Lokomat® training (Table 2). Almost 30% of the patients started their training within the first month post event.

**Table 2:** Time between lesion and first Lokomat® training

Patient groups	Proportion of patients [%]				
	0–1 month	1–3 months	3–6 months	6–12 months	> 12 months
Stroke (*n* = 71)	28%	30%	15%	8%	18%
SCI (*n* = 92)	29%	18%	15%	10%	27%
TBI (*n* = 28)	29%	14%	29%	7%	21%

*Abbreviations*: *SCI* spinal cord injury, *TBI* traumatic brain injury. Number of missing observations: stroke, *n* = 61; SCI, *n* = 2; TBI, *n* = 11

Concerning clinical characteristics (Fig. 3), the largest number of patients with CP had a spastic bilateral CP and a GMFCS level III or IV. A small majority of the patients with stroke had experienced an ischemic rather than a hemorrhagic lesion. The lesion of patients with SCI was predominantly of traumatic origin (72%), had resulted in most patients in an AIS C, and the neurological level of lesion was most frequently diagnosed at cervical 4 (Fig. 3).

As we plan detailed analyses on the amount of training for future studies, we refrained from any statistical analyses in the current paper. Initial results show that in general the intensity of training seems to increase (i.e. longer training duration, larger distances covered, higher walking velocities and less supportive force from the Lokomat®) from the first to the final Lokomat® training session (Table 3).

**Table 3:** Training parameters (mean ± standard deviation) at onset and end of Lokomat® training period

Patients		Duration walking time per session [min]		Distance walked per session [m]		Average walking speed [km/h]		Guidance Force [%]^a^		Number of training sessions
	GMFCS	Onset	End	Onset	End	Onset	End	Onset	End	
Cerebral Palsy	II	24.7 ± 9.3	31.6 ± 10.7	555 ± 282	918 ± 320	1.12 ± 0.53	1.45 ± 0.63	85.1 ± 14.5	69.4 ± 20.4	27.2 ± 28.8
	III	23.4 ± 12.2	33.3 ± 11.6	524 ± 355	917 ± 401	1.13 ± 0.51	1.41 ± 0.59	83.1 ± 17.4	73.1 ± 18.8	23.4 ± 17.2
	IV	24.2 ± 9.9	33.9 ± 10.3	522 ± 295	980 ± 373	1.25 ± 0.38	1.68 ± 0.42	84.3 ± 14.6	74.6 ± 16.0	21.8 ± 13.7
Stroke	Ischemic	16.7 ± 9.0	29.3 ± 10.7	409 ± 258	886 ± 372	1.14 ± 0.57	1.42 ± 0.66	92.9 ± 16.4	85.4 ± 16.4	16.2 ± 14.5
	Hemorrhagic	16.1 ± 6.8	29.3 ± 9.9	386 ± 198	858 ± 374	1.05 ± 0.60	1.26 ± 0.64	90.0 ± 23.4	81.4 ± 19.5	17.9 ± 17.9
Spinal cord injury	AIS C	16.7 ± 6.6	24.0 ± 9.5	423 ± 247	759 ± 368	1.65 ± 0.44	1.88 ± 0.50	98.1 ± 5.0	84.2 ± 19.6	10.3 ± 14.8
	AIS D	19.1 ± 3.1	22.2 ± 4.5	536 ± 124	700 ± 113	1.55 ± 0.52	1.77 ± 0.58	98.0 ± 2.7	92.2 ± 10.3	30.5 ± 60.3
Traumatic brain injury		15.4 ± 7.7	21.8 ± 13.6	350 ± 162	624 ± 428	1.14 ± 0.68	1.37 ± 0.73	85.0 ± 22.4	79.3 ± 22.5	24.1 ± 29.4

*Abbreviations*: *GMFCS* Gross Motor Function Classification System, *AIS* ASIA Impairment Scale. ^a^Presented for left leg

**Fig. 5:**
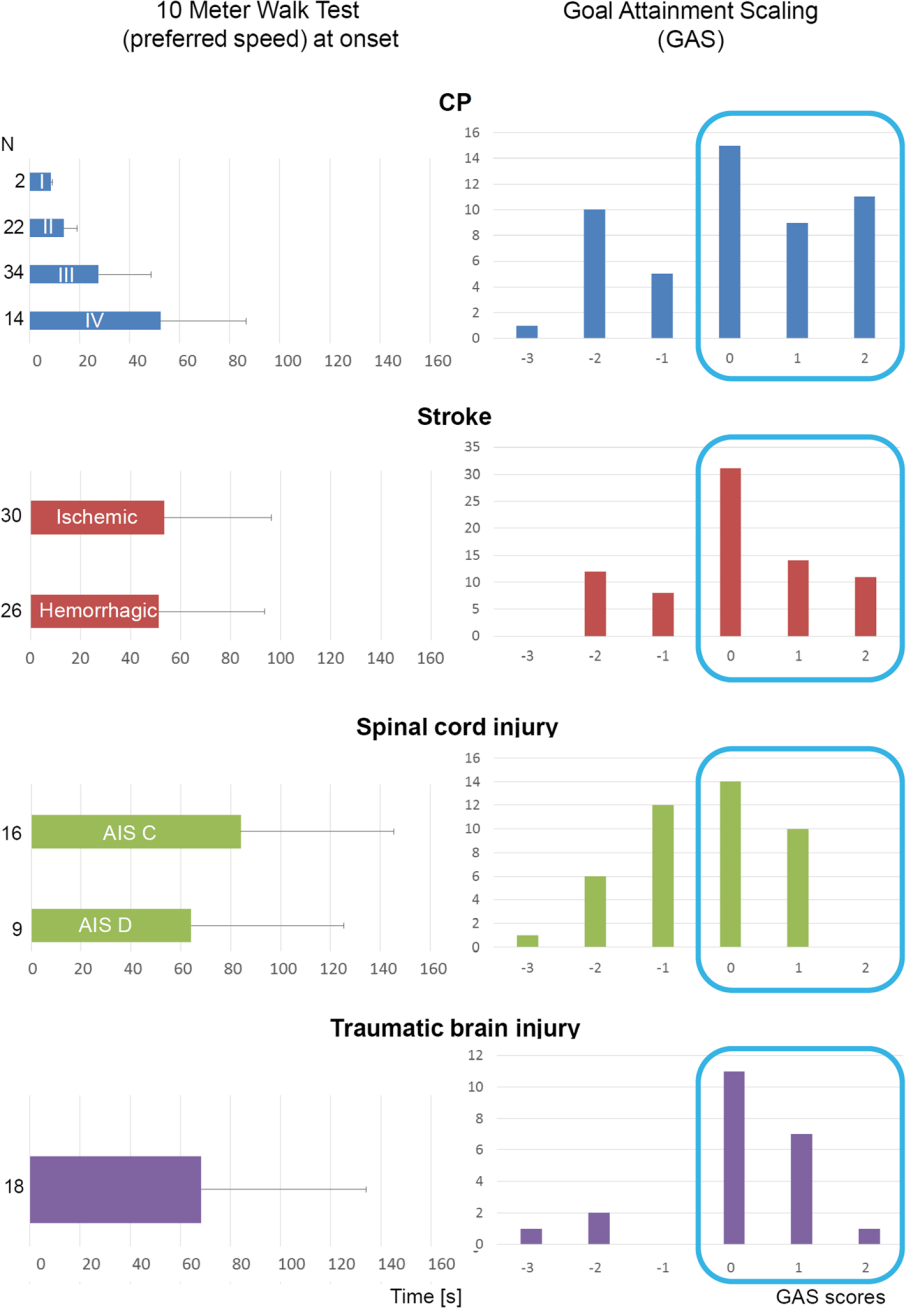
Clinical outcome measures. On the left are the average and SD times (in seconds) presented that the patients need to walk 10 m at onset of Lokomat® training. For some patient groups, results are separated according to severity grades (e.g. the Gross Motor Function Classification Systems levels I, II, III or IV for patients with CP or the ASIA Impairment Scales C and D for patients with spinal cord injury) or etiology of stroke (ischemic versus hemorrhagic). Displayed at the right are the Goal Attainment Scores. Despite poor initial walking function, many patients achieved their goals (Goal Attainment Scores 0, 1 or 2; encircled in blue)

Finally, we show initial results of the 10MWT performed at onset of the Lokomat® intervention (Fig. 5). The time needed to walk 10 m is considerable and seems to be longer for patients with higher severity grades.

Despite poor initial walking function, many patients achieve their goals, as indicated by the GAS. In general, only single patients deteriorate (GAS score − 3), some remain at their initial level (GAS score − 2), and several improve, but less than expected (GAS score − 1). Most patients seem to achieve their goal (GAS score 1) or somewhat (GAS score + 1) or even much more (GAS score + 2) than expected. The percentages of patients that achieve their goal (encircled in blue. Figure 5) amounts to 69% (CP), 74% (stroke), 56% (SCI) and 83% (TBI).

## Discussion

The ARTIC network was successful at getting a number of centers to contribute data without the need for a financial incentive. The network was able to amass a relatively large dataset in a relatively short time. The data collected included subject characteristics, outcomes, and training information.

We addressed the first two aims of this paper, to introduce the ARTIC network to the clinical and research community and present first data on the characteristics of included patients, in the previous paragraphs. We will discuss below the characteristics of the patients included in the ARTIC database by comparing them with those published in epidemiological and interventional studies, in line with the third aim of this study.

### Cerebral palsy

Compared to epidemiological studies, the ARTIC database contains an overrepresentation of children with bilateral spastic CP (86%) and an underrepresentation of children with unilateral spastic CP (4%). The proportion of children with bilateral spastic CP varies between countries (e.g. Denmark 51% and Norway 43%, [37]). Spastic bilateral CP was found in 45.7% of the 3′948 children from 14 Surveillance of Cerebral Palsy in Europe (SCPE) registers in Europe [38] and in 49.2% of the 451 children with CP from the Autism and Developmental Disabilities Monitoring (ADDM) Network in four areas in the USA [39]. The proportion of children with unilateral spastic CP varies between 28.2% and 40% (Denmark: 37%, Norway: 40%, [37]; SCPE: 39.2%, [38]; ADDM Network: 28.2%, [39]. In general, children with an ataxic or dyskinetic (dystonic or chorea-athethotic) CP are relatively rare (e.g. [37–39]), and this is in line with the small numbers in our database.

The ARTIC database shows an overrepresentation of children with GMFCS levels III and IV (each 39%, see Fig. 3) compared to epidemiological numbers. Christensen et al. [39] reported that GMFCS levels III and IV accounted for 12.3% and 16.8%, respectively. In children with bilateral spastic CP, these percentages were higher and amounted to 15.3% (level III) and 21.2% (level IV). They further mention that ‘nearly all children with unilateral spastic CP walked independently (96.6%) compared with less than half of those with bilateral spastic CP’ (45.4%). The cause of the ‘overrepresentation’ of children with a bilateral spastic CP with GMFCS levels III and IV in the ARTIC database is most likely due to the clinical reasoning underlying the selection of patients treated with the Lokomat®. Their walking ability is moderate to severely affected, but still, the goal is to achieve a certain level of walking, which would exclude children with GMFCS level V.

The authors are aware of only two published randomized controlled Lokomat® trials in children with CP. Druzbicki et al. [7] aimed at investigating changes in temporospatial and kinematic gait parameters in children with spastic diplegia. The children were between 6 and 13 years old, had spastic diplegia, the ability to independently stand and walk or walk with assistance, and a GMFCS level II or III. They excluded children with disorders of higher mental functions, Lokomat® related exclusion criteria, and those who were treated with botulinum toxin during the last 6 months and treated surgically within the past year. While the participants should perform 20 sessions a 45 min, the actual training dosage was not reported. Wallard et al. [8] investigated the effect of Lokomat® training on the dynamic equilibrium control during walking. They included 30 children with bilateral spastic CP and jump gait, GMFCS level II, aged 8 to 10 years, and the ability to independently stand and walk or walk with assistance (e.g. walking stick). They should perform 20 sessions (maximally 40 min of walking time), but did not report the actual training dosage. It seems that the patients in the Wallard et al. [8] study were less severely affected compared to the patients in the ARTIC database, as their children would need on average about 11.8 s to complete the 10MWT (initial walking speed of 0.85 m/s). While also the patients in the study by Druzbicki et al. [7] seem at first sight less affected (e.g. GMFCS levels II and III), these patients would have needed on average about 29 s to perform a 10MWT (initial walking speed of 0.35 m/s.), which is more than the average GMFCS level III patients would need in the ARTIC (see Fig. 5).

Due to these differences, it remains difficult to estimate to what extent the results from such interventional studies can be generalized to the patients who receive their Lokomat® training clinically.

### Stroke

The Global Burden of Diseases, Injuries, and Risk Factors (GBD 2013) study presented an update on the global burden (data from 188 countries) of ischemic and hemorrhagic stroke between 1990 and 2013 [40]. If we compare the patients’ characteristics of the ARTIC database with the 2013 numbers from developed countries from that paper, we see an overrepresentation of patients with hemorrhagic stroke in the ARTIC database (43% ARTIC versus around 20% worldwide). This might be explained by the finding that patients with a hemorrhagic stroke are often more severely affected than patients with ischemic stroke. Furthermore, the number of children with stroke is overrepresented in the ARTIC database (Fig. 4), which can be explained with the relatively high number of participating tertiary pediatric rehabilitation centers in our network. While we observe a peak in patients aged 60 to 70 years old (Fig. 4), the worldwide incidence and prevalence of patients after stroke is higher in patients aged 70 years and older [40]. A discussion of this difference is speculative, but we suggest that patients of higher age, with multiple co-morbidities and poorer general health condition, will be less frequently referred for robot-assisted training or are cared for in nursing homes. The equal distribution of females versus males in our database is in line with the gender distribution worldwide [41] or hospitalization rates in the USA [42].

Many randomized controlled trials with the Lokomat® have been performed in patients with stroke (e.g. [43–50]). Results from these studies have been summarized in systematic reviews (e.g. [2, 51]) and recommendations have been made in national treatment guidelines (e.g. [52]). Only general observations can be made at this time comparing available data.

Most studies included patients within the first year after stroke, except for, for example, Kelley et al. [46] and Bang and Shin [43] who also included chronic patients. The time after lesion should be taken into consideration, as in subacute patients spontaneous neurological recovery might also contribute to the observed functional improvements.

All studies included only patients with a first-ever stroke and most studies excluded patients with accompanying orthopedic or neurological diagnoses or multiple medical problems. While most Lokomat® sessions lasted around 45 min, the number of sessions varied between these trials: 12 [50], 15 or 30 [47], 16 [49], 18 [48], 20 [43, 45], 24 [44] or 40 [46]. As the average age was around 50 years old (e.g. [49]) or older, it is likely that many patients had to be excluded due to co-morbidities. This is in line with the practical observation that patient recruitment in the field of stroke research for randomized studies takes time. Therapists working with the Lokomat® should therefore not be disappointed when progress in gait training in their patients with stroke is less as reported in randomized trials, as these patients might differ on several aspects relevant for relearning to walk.

### Spinal cord injury

The National SCI Statistical Center estimates that the annual incidence of SCI in the US approximates 54 cases per million inhabitants [53]. The average age is currently 42 years (distribution of age at onset is shown in Fig. 4), and about 80% of the new cases are males. Both numbers are more or less in line with those reported in our data. At onset of rehabilitation, the distribution of the AIS was according to the National SCI Statistical Center: AIS A, 40.5%; AIS B, 12.2%; AIS C, 18.2%; AIS D, 29.0% and AIS E, 0.0% (data from 4′452 patients). We have considerably fewer patients in ARTIC with AIS A and considerably more with AIS C. One simple explanation is that most patients with AIS A do not have the goal of achieving independent walking and, therefore, do not receive Lokomat® training. Patients with AIS C are severely impaired in gait, but have potential to improve and can therefore be considered a target group for Lokomat® therapy. While the National SCI Statistical Center reports level of lesion only at discharge, the distribution of the neurological level of lesion is well comparable to ours (Fig. 3f).

Various randomized controlled Lokomat® studies in patients with SCI have also been published. A recent systematic review reported studies on pediatric and adult patients with SCI [54]. Several randomized Lokomat® studies have been performed in patients with SCI showing that also in these patients the number of studies is growing steadily [55–60]. In these studies, the (average) number of training sessions varied considerably: 16 [58], 34 [60], around 40 [55, 56, 59] or even 60 [57].

When comparing the characteristics of the patients in the ARTIC database with the patients in these studies, most studies included patients with AIS C or D. None included patients with AIS A and only some (e.g. [60]) included patients with AIS B. Some patients in the ARTIC database are diagnosed as AIS A or B. At first sight, it might not make clinical sense to include patients with a motor complete lesion (AIS A and B) in a driven gait orthosis to improve walking, but one should also think of the level of lesion that could play a role. While most studies excluded patients with signs of lower motor neuron lesions, one study investigated differences between patients with upper versus lower motor neuron lesions specifically [56]. Looking at the level of lesion of most patients in the ARTIC database, there will be only a few patients with lower motor neuron lesions undergoing Lokomat® training. Most studies included patients with both traumatic and stable non-traumatic lesions and this is in line with the patients, which receive Lokomat® training in the ARTIC network. In line with the previous discussion on age and multiple medical issues, the average age of the patients with SCI was in most studies between 40 and 50 years (e.g. [55–57]), while it exceeded 50 years in other studies (e.g. [58, 59]). In the ARTIC database, 43% of the patients with SCI are 50 years or older. As most randomized studies reported that patients should have no other neurological, orthopedic or cardiovascular issues, we can assume that also for these studies inclusion and exclusion criteria resulted in a particular patient population that might not entirely resemble the patients with SCI that receive Lokomat® therapy in a clinical setting.

### Traumatic brain injury

According to the Traumatic Brain Injury Model System (TBIMS) [61], the distribution of gender of patients with TBI who received inpatient rehabilitation in the USA between 1989 and 2015 was 26% females and 74% males (similar number for 2016). These numbers are comparable to our data (23% females and 77% males). The average age of patients with TBI in the TBIMS database is 41.4 years (*n* = 14′624), which appears older compared to the age of the patients with TBI included in our data. This is likely caused by differences in the inclusion criteria, as patients should be 16 years or older to be included in the TBIMS database, while we also include(d) younger children and adolescents. According to the Centers for Disease Control and Prevention (CDC), those most likely to sustain a TBI are children aged 0 to 4 years, older adolescents aged 15 to 19 years, and adults aged 65 years and older [62]. While our data also show the highest peak in adolescents aged 10–20 years, we find no peak in children below 10 years. We can easily explain this lack of agreement by differences in epidemiology (0–4 years) versus Lokomat® training inclusion criteria (older than 4 to 5 years). Unlike epidemiological data, we find no peak in patients aged 65 years and older. Perhaps similar to our suggestion for the elderly patients with stroke, physicians might refer these patients less frequently for robot-assisted training due to the higher age, multiple co-morbidities and poorer general health condition.

To our knowledge, literature on the use of the Lokomat® in patients with TBI is rare and we are familiar with only one randomized controlled trial [6]. The chronic patients aged 18 years or older participated in 18 sessions and walked at onset on average with a speed of around 0.37 m/s (estimated from the figure). As this corresponds to about 27 s for the 10MWT, these patients seem better compared to the patients with TBI in ARTIC who needed almost 70 s to complete the 10MWT (Fig. 5). Importantly, the ARTIC database uses few characteristics to describe the patients with TBI. This is a limitation that should be resolved if we intend to improve our knowledge on the applicability of Lokomat® training for this patient group.

In summary, these paragraphs show that the characteristics of the patients included in ARTIC fitted in general with epidemiological data. We can explain certain deviations from epidemiological numbers due to particular patient selection for undergoing Lokomat® training and the high number of participating pediatric centers. Furthermore, while patients included in randomized controlled interventional trials have to fulfill many inclusion and exclusion criteria, the only selection criteria applying to patients in the ARTIC database are those related to the use of the Lokomat®, which make it difficult to generalize findings from randomized trials to patients treated on a daily basis.

In the introduction, we mentioned that such narrow in- and exclusion criteria of interventional studies might limit the ecological validity, as results only apply to a select subgroup of patients. While this could allow for better targeting of specific treatments to specific patients, it limits its generalization. Improving our knowledge of biomarkers that could differentiate between responders and non-responders, is an important area of research. For example, in a recent publication, participants from the Stroke Recovery and Rehabilitation Roundtable published a paper in which they made recommendations on when and where to include biomarkers derived from imaging or neurophysiological techniques for advancing practice and research [63]. While we agree that rehabilitation in the future could be more effective for patients identified as responders, it should be noted that ‘ready for use’ stroke biomarkers are currently lacking for most functional domains [63]. Furthermore, it remains unanswered how therapists should treat ‘non-responding’ patients in their daily practice. Likely, therapists will apply task-specific, repetitive, intensive exercises focusing on improving major impairments and limitations of the individual patient. These patients might show less or slower progress compared to patients identified as responders. Our network aims to contribute to these developments, as we might identify patients who might respond better to RAGT or recognize characteristics of interventions that prove more effective than others.

### Training dosage and parameters

While the number of training sessions varied between the randomized trials that we reported, they vary considerably in our database, as indicated by the large standard deviations. For children with CP, the average number of training sessions in ARTIC slightly exceeds those reported in the clinical trials (*n* = 20). The average number of sessions for patients with stroke (16 to 18 sessions) seems in line with the majority of the randomized trials that we referenced to and is in general relatively low. It is possible that natural recovery and increased responsiveness leads to shorter duration of Lokomat® training. For patients with SCI, we notice a low mean number of sessions (*n* = 10) for patients with AIS C and around 30 sessions for patients with AIS D. Despite that we would consider patients with AIS C a typical target group for Lokomat® training, it is possible that lack of rapid gains in these patients led to less training or that these patients might have changed to over-ground training conditions as soon as they could bear weight and initiate stepping movements. Finally, patients with TBI in ARTIC receive on average slightly more sessions (*n* = 24) compared to patients who participated in the (only) randomized trial (*n* = 18). While the number of sessions is an important measure for future analysis on dosage-response relationships, discussions on its differences between randomized studies and ARTIC or between patient groups are limited by practical issues such as insurance companies who might finance only a specific number of sessions, irrespective of severity of limitations.

Interestingly, most randomized studies do not report detailed information on training dosage and parameters (one positive exception is e.g. [60]). Most studies stated that *x* sessions of *y* minutes of training with the Lokomat® or control intervention were performed. They lack an accurate listing of the actually achieved number of training sessions, pure training times, and training parameters such as exact duration of walking, walking speed or guidance force. Such information is valuable, as it would allow in combination with functional outcome measures to estimate the dosage needed to achieve a particular functional improvement and make recommendations for training protocols. For future studies, it would be of interest that authors report the interventions into more detail. Authors could adhere to the recommendations formulated in the Template for Intervention Description and Replication (TIDieR) checklist [64]).

When looking at Table 2, it seems that especially the walking duration, the distance walked, and the average walking speed increase considerably during the training sessions. While we assume that this is due to the improved ability of the patient to participate in training, the Lokomat® parameters could be influenced by other issues we cannot fully determine from the data. Compared to the other parameters, the guidance force seems to change less from onset to end. Guidance force can be adjusted from 100% (this is impedance control, which corresponds to a position-controlled mode) to 0%. At 0% the Lokomat® will not provide support for the patient’s movements and should only compensate for robotic dynamics (gravity and Coriolis forces) but not for inertia (for an overview see [65]). At 100% guidance, no deviation is possible from a predetermined movement trajectory for the hip and knee joints and, theoretically, patients do not need to participate actively. In healthy participants, walking under 100% guidance force generally reduces muscle activity, but even then, muscles show phased activity, indicating that participants still actively controlled the gait movements [66]. In children with neuromotor disorders, walking under 100% guidance force results in a more physiological activation of leg muscles compared to unassisted treadmill walking [67] or to Lokomat® training with other control strategies that allow more variability [65]. We might therefore assume that therapists prioritize a physiological walking pattern and rather adjust parameters like duration or speed to increase the intensity of the training, while maintaining a physiological pattern. However, more detailed analyses including additional parameters and multiple time points during the training program are needed to verify this assumption.

### Creating the database – Lessons learned

Many factors led to the success of bringing these data together. Foremost was leadership and support of the company. While no sites were paid to enter data, many processes were put in place to make this as easy as possible. In addition, the company supported meetings and produced the minutes. The company did not have a vote on critical decisions made by the network nor on the contents of the ARTIC network’s scientific output. This effort represents an example of industry clinical partnership that has the ability to benefit the clinical and research community.

Another critical factor was a steady and concerted effort to not make data collection onerous. While researchers desire in general to collect more data, clinical partners might stop participating if data collection might require too much time. It is clearly a tradeoff, as more data would allow analyses that are more detailed. The ARTIC network hopes they struck the right balance, and while initial results seem positive, further analyses are needed.

Finally, this work was made possible through numerous people at each site who believed in the effort and volunteered their time.

### Limitations

Like most networks, it takes considerable time not only to increase the numbers but also to improve the quality of the data (e.g. completeness, standardization, accurate reporting). We, therefore, maintain a continuous effort to train personnel and improve the database content. Nevertheless, we initially had missing data. Furthermore, from time to time, we updated the database. New, more detailed, data can be collected but is missing for those patients who received their Lokomat® training during an earlier phase. We, therefore, presented in this study only data of patient groups that are more complete. We will perform future evaluations for specific patient groups to get information that is more detailed in terms of patient criteria, changes in training parameters and functional progress, and goal attainment.

Concerning goal attainment, it is promising that most patients achieved their goals, but we need to consider that many patients also received additional conventional interventions besides Lokomat® therapy.

Furthermore, due to the pragmatic nature of this data collection effort, all patients undergo Lokomat® training and we do not have a control group. Also, we will not be able to collect data in similar detail as, for example, randomized controlled clinical trials. This has, of course, its limitations concerning our research questions. Nevertheless, if additional outcomes are desired, the network provides an excellent basis to start such a project. Experienced assessors already assess many measures in a standardized way, and we could add additional project specific outcomes if financial resources are available.

## Conclusions

As the degree of performance improvement depends on the amount of practice, rehabilitation robots have the potential to provide such intensive therapies. Importantly, the ARTIC network assesses the application and changes in function, activities and patient-relevant tasks in the real neurorehabilitative environment. While patients included in randomized controlled interventional trials have to fulfill many inclusion and exclusion criteria, the only selection criteria applying to patients in the ARTIC database are those related to the use of the Lokomat®. Indeed, while some patient characteristics differed from epidemiological data, mainly due to particular patient selection for undergoing Lokomat® training, most characteristics fitted with epidemiological data.

This network offers a unique opportunity to investigate the implementation, application, and effectiveness of rehabilitation technologies. Within the network, clinical practice and research are strongly interconnected. There is a continuous exchange of knowledge and expertise between researchers and therapists. The network offers the opportunity to develop standardized protocols and guidelines for the application of robotic technologies. Such guidelines could achieve strong international acceptance as experienced rehabilitation specialists working in internationally renowned centers develop them.

## Abbreviations

10MWT: 10-Meter Walk Test
ADDM: Autism and Developmental Disabilities Monitoring
AIS: ASIA Impairment Scale
ARTIC: Advanced Robotic Therapy Integrated Centers
CDC: Centers for Disease Control and Prevention
CP: Cerebral Palsy
FIM: Functional Independence Measure
GAS: Goal Attainment Scaling
GBD: Global Burden of Diseases, Injuries, and Risk Factors
GMFCS: Gross Motor Function Classification System
ICF: International Classification of Functioning, Disability and Health
IRB: Institutional Review Board
MAS: Modified Ashworth Scale
MS: Multiple Sclerosis
N: number
RAGT: Robot-Assisted Gait Training
SCI: Spinal Cord Injury
SCPE: Surveillance of cerebral palsy in Europe
TBI: Traumatic brain injury
TBIMS: Traumatic Brain Injury Model System
WeeFIM: Functional Independence Measure for Children
