# Correction to: Advanced Robotic Therapy Integrated Centers (ARTIC): an international collaboration facilitating the application of rehabilitation technologies

**DOI:** 10.1186/s12984-018-0378-7

**Published:** 2018-05-08

**Authors:** Hubertus J. A. van Hedel, Giacomo Severini, A. Scarton, A. O’Brien, T. Reed, D. Gaebler-Spira, T. Egan, A. Meyer-Heim, J. Graser, K. Chua, D. Zutter, R. Schweinfurther, J. C. Möller, Liliana P. Paredes, A. Esquenazi, S. Berweck, S. Schroeder, B. Warken, A. Chan, A. Devers, J. Petioky, Nam-Jong Paik, W. S. Kim, P. Bonato, M. Boninger, ARTIC network, E. Fabara, C. Adans-Dester, J. O’Brien Murby, L. Laliberte, G. Revivo, S. Lee, T. Toczylowski, K. F. Chan, S. K. Wee, P. H. Lim, W. S. Lim, J. Y. Y. Wang, W. K. Lee, C. N. Ong, C. H. Ong, C. C. Pereira, S. Y. Lee, A. Dewor, M. Urban, T. Aurich, A. Lucic, T. Nastulla, K. Badura, J. Steinbichler, M. Ji, Y. Oh, Rocco Salvatore Calabrò, L. van Hiel, M. Spiess, L. Lünenburger, G. Colombo, I. Maier

**Affiliations:** 1 Rehabilitation Center for Children and Adolescents, University Children’s Hospital Zurich, Mühlebergstrasse 104, CH-8910 Affoltern am Albis, Switzerland; 2 Department of Physical Medicine and Rehabilitation, Harvard Medical School, at Spaulding Rehabilitation Hospital, Charlestown, MA USA; 3 University College Dublin, Dublin, Ireland; 4 Acute Neurological Rehabilitation Unit, Wellington Hospital, London, UK; 5 Shirley Ryan AbilityLab, Chicago, USA; 6 Rehabilitation Center for Children and Adolescents, University Children’s Hospital Zurich, Mühlebergstrasse 104, CH-8910 Affoltern am Albis, Switzerland; 7 Tan Tock Seng Hospital Rehabilitation Centre, Singapore, Republic of Singapore; 8 Rehaklinik Zihlschlacht, Center for Neurological Rehabilitation, Zihlschlacht, Switzerland; 9 Department of Physical Medicine and Rehabilitation, MossRehab, Philadelphia, USA; 10 Clinic for Neuropediatrics and Neurological Rehabilitation, Epilepsy center for children and adolescents, Schön Klinik Vogtareuth, Vogtareuth, Germany; 11 Paediatric Neurology, Developmental Medicine and Social Paediatrics, Ludwig Maximilian University, Hauner Children’s Hospital, Munich, Germany; 12 Sheltering Arms Physical Rehabilitation Center, Richmond, USA; 13 Rehabilitation Centre Kladruby, Kladruby, Czech Republic; 14 Department of Rehabilitation Medicine, Seoul National University Bundang Hospital, Seongnam, Republic of Korea; 15 Department of Physical Medicine and Rehabilitation, University of Pittsburgh and VA Pittsburgh Health Care System, Pittsburgh, USA

## Correction

The original article [1] contains a small mistake concerning the ARTIC Team members mentioned in the Acknowledgements. The team member, Rocco Salvatore Calabrò had their name presented incorrectly. This has now been corrected in the original article.
